# Impact of Hunger, Satiety, and Oral Glucose on the Association Between Insulin and Resting-State Human Brain Activity

**DOI:** 10.3389/fnhum.2019.00162

**Published:** 2019-05-14

**Authors:** Arkan Al-Zubaidi, Marcus Heldmann, Alfred Mertins, Georg Brabant, Janis Marc Nolde, Kamila Jauch-Chara, Thomas F. Münte

**Affiliations:** ^1^Department of Neurology, University of Lübeck, Lübeck, Germany; ^2^Institute of Psychology II, University of Lübeck, Lübeck, Germany; ^3^Institute for Signal Processing, University of Lübeck, Lübeck, Germany; ^4^Department of Internal Medicine I, University of Lübeck, Lübeck, Germany; ^5^Department of Psychiatry and Psychotherapy, Christian-Albrechts-University, Kiel, Germany

**Keywords:** resting state fMRI, hunger, satiety, glucose administration, fALFF, insulin levels

## Abstract

To study the interplay of metabolic state (hungry vs. satiated) and glucose administration (including hormonal modulation) on brain function, resting-state functional magnetic resonance imaging (rs-fMRI) and blood samples were obtained in 24 healthy normal-weight men in a repeated measurement design. Participants were measured twice: once after a 36 h fast (except water) and once under satiation (three meals/day for 36 h). During each session, rs-fMRI and hormone concentrations were recorded before and after a 75 g oral dose of glucose. We calculated the amplitude map from blood-oxygen-level-dependent (BOLD) signals by using the fractional amplitude of low-frequency fluctuation (fALFF) approach for each volunteer per condition. Using multiple linear regression analysis (MLRA) the interdependence of brain activity, plasma insulin and blood glucose was investigated. We observed a modulatory impact of fasting state on intrinsic brain activity in the posterior cingulate cortex (PCC). Strikingly, differences in plasma insulin levels between hunger and satiety states after glucose administration at the time of the scan were negatively related to brain activity in the posterior insula and superior frontal gyrus (SFG), while plasma glucose levels were positively associated with activity changes in the fusiform gyrus. Furthermore, we could show that changes in plasma insulin enhanced the connectivity between the posterior insula and SFG. Our results indicate that hormonal signals like insulin alleviate an acute hemostatic energy deficit by modifying the homeostatic and frontal circuitry of the human brain.

## Highlights

- Brain areas relevant for ingestive behavior are modulated as a function of hunger/satiety.
- Metabolic state, insulin levels and glucose administration interact in their effects on brain activation patterns.
- FALFF is a reliable index of spontaneous brain activity.

## Introduction

Food ingestion and energy homeostasis are regulated by central nervous pathways (Schwartz and Porte, 2005; Lam, 2010; Suzuki et al., 2010) and modulated by endocrine signals (Gao and Horvath, 2008). Insulin is one of the hormones that is part of a negative feedback loop that ensures balanced energy homeostasis (Schwartz et al., 2000; Schwartz, 2001; Obici et al., 2002). Of note, both intraventricular injection and intranasal administration of insulin decreases food intake and body weight in rodents (Chavez et al., 1995; Brown et al., 2006) and humans (Benedict et al., 2008). In contrast, inactivation of insulin leads to opposite effects (McGowan et al., 1990; Brüning et al., 2000).

Task-based functional magnetic resonance imaging (fMRI) has been used to study the impact of insulin and glucose on brain responses to food-related stimuli under different homeostatic conditions on the network level (Woods et al., 1998; Liu et al., 2000) linking plasma glucose (Kroemer et al., 2013b) and fasting insulin levels (Wallner-Liebmann et al., 2010) to neural activity changes in the hypothalamus, thalamus, amygdala, insula, and superior frontal cortex, brain sites assumed to regulate appetite-related eating behavior (Kullmann et al., 2013). According to Smitha et al. (2017), task-based designs focus on a small fraction of the brain’s overall activity only. To avoid these disadvantages in the present investigation, we used resting-state fMRI in order to reveal potential links between whole brain activity and insulin and glucose levels in different metabolic states.

Resting-state fMRI (rs-fMRI) is a task-free paradigm in which participants do not perform any specific task (Biswal et al., 1995; van den Heuvel and Hulshoff Pol, 2010; Lee et al., 2013). Several studies using rs-fMRI revealed associations of insulin or glucose levels with the functional connectivity (FC) of particular brain networks or brain sites that are related to homeostatic regulation but not without contradictions. Some studies reported an increase of resting-state FC within the limbic system in response to the administration of 75 g oral glucose after overnight fasting. This increase in FC was positively correlated with plasma insulin levels (Kullmann et al., 2012; Wölnerhanssen et al., 2015). Conflicting results were found by Page et al. (2013) using MRI-cerebral blood flow (CBF) during rest: here, changes of insulin levels were negatively associated with changes of CBF signals in the caudate and the putamen in response to glucose administration. Similarly, changes in plasma insulin levels in response to a meal after overnight fasting were negatively correlated with changes of CBF signals in the insula and prefrontal cortex (Tataranni et al., 1999). Additionally, the FC between the posterior insula (PINS) and superior frontal gyrus (SFG) under hunger conditions was partially moderated by the plasma glucose levels, indicating that the PINS connectivity depends on the homeostatic energy deficit caused by fasting (Wright et al., 2016). Furthermore, a study with experimentally induced hypoglycemia reported increased FC of the default mode network (DMN) with posterior cingulate cortex (PCC) and decreased FC of insula, superior and inferior frontal gyri with temporal networks, basal ganglia, and cerebellum in healthy subjects (Bolo et al., 2015). The inconsistencies reported here might be related to the different experimental paradigms and neuroimaging modalities. In most of these studies, FC parameters were computed by using seed-based analysis or independent component analysis (ICA) to define brain networks of interest or to decompose the brain into multiple networks, respectively. This means that FC studies rely on the correlations and ignore the changes in regional brain activity under different metabolic conditions. However, it has been shown that there is a linear relationship between the amplitude of the blood-oxygen-level-dependent (BOLD) signal and brain metabolism (Tomasi et al., 2013). Therefore, in our study, we used the amplitude of the rs-fMRI signal to investigate the sensitivity of resting-state brain activity (Zou et al., 2008) to changes in metabolic states. A critical question of the present search is, whether or not the changes in the amplitude of the BOLD signal of specific brain regions are associated with changes in hormonal signals, such as insulin.

To clarify some of the issues discussed in the previous paragraph, we investigated: (i) whether changes in brain regions linked to hypoglycemia, such as the DMN, are modulated by insulin and glucose or different metabolic states; and (ii) whether changes in activity of specific brain regions, such as hypothalamus and insula, are modulated by changes of blood insulin or glucose levels after glucose administration. In the present investigation, we used a voxel-wise frequency-domain approach to measure whole brain activity by using amplitude (intensity) values of BOLD signals called fractional amplitude of low-frequency fluctuation (fALFF) for each individual per condition (Zou et al., 2008). The fALFF allow us to study the local spontaneous brain activity across the whole brain based on the magnitude of the BOLD signal in the low-frequency range (Zou et al., 2008; Egorova et al., 2017) which is higher in gray than in white matter (Zuo et al., 2010). The fALFF approach efficiently suppresses non-specific signal components, such as physiological noise (Zou et al., 2008; Cole et al., 2010). Other studies have observed that fALFF is associated with body mass index (BMI) after intranasal insulin application (Kullmann et al., 2013).

In a recent rs-fMRI study (Al-Zubaidi et al., 2018), we examined the activity and connectivity brain responses to the interaction of metabolic state (hungry vs. satiated) and glucose administration (before vs. after administration of 75 g of oral glucose). In that study, we used multimethod rs-fMRI approaches to identify brain activation patterns that are associated with changes in metabolic states and caloric intake. We showed that in contrast to other voxel-wise analyses like regional homogeneity or degree of centrality, fALFF is a more sensitive metric for identifying differences in the resting brain activity, for example, the amplitude of the SFG and PCC were increased after oral glucose treatment and in hunger conditions, respectively. However, our previous study focused on the effect of glucose treatment solely on brain activity and connectivity without taking the effect of plasma glucose and insulin levels into account when analyzing neuroimaging data (Al-Zubaidi et al., 2018). To get a better insight into hormone-dependent brain activity at rest, in the present study we used multiple linear regression analysis (MLRA) with covariates (plasma insulin and glucose levels) to investigate the dependencies between brain activity, measured as the amplitude of the BOLD signal, and hormone levels. By manipulating metabolic state (hunger/satiety) and glucose administration (before and after 75 g of oral glucose) in a factorial design, we tested the hypothesis that brain areas involved in homeostatic regulation are modulated by peripheral circulating insulin and glucose levels (Williams et al., 2000; Figlewicz, 2003; Zanchi et al., 2017).

## Materials and Methods

### Participants

We investigated 24 healthy male volunteers aged 20–30 years (mean ± SEM: 24.5 ± 0.6 years) with a BMI of 20–25 kg/m^2^ (mean ± SEM in kg/m^2^: 23.4 ± 0.3) recruited from the local university community. All participants underwent a medical interview before participating in this study and examination assessing general health, medicines, mood, blood glucose concentration and cognitive disorders. More details on the exclusion criteria of participants can be found in Al-Zubaidi et al. (2018). Within 4 weeks before and during the trials, subjects were instructed not to participate in other studies or to donate blood. The study was approved by the ethics committee of the University of Lübeck and carried out under the Declaration of Helsinki (2000). Before participation, each participant gave written informed consent.

### Experimental Design

Each subject was investigated twice, once while being in a hungry (36 h fasting) and once while being in a satiated state (standardized eating, three meals/day for 36 h). The order of the two sessions was counterbalanced across participants, with a break of at least 1 week between sessions. To control physical activity and food intake, subjects stayed at the sleep laboratory of the Department of Psychiatry of the University Hospital of Lübeck (UKSH Lübeck) for 36 h in both conditions. At the end of each 36 h stay, the functional MRI session took place. Each MRI session started with the first resting state recording. Subjects were removed from the scanner to drink a solution containing the equal of 75 g of glucose (determined by a 300 ml mixture of mono and oligosaccharides; ACCU-CHEK^®^ Dextro^®^ O.G-T., Roche, Grenzach-Wyhlen, Germany). To avoid an impact of any circadian variations the timing of the glucose drink was kept constant and started each time at 01:25 pm. Twenty minutes after glucose intake, the second resting state fMRI was recorded.

**Figure 1:**
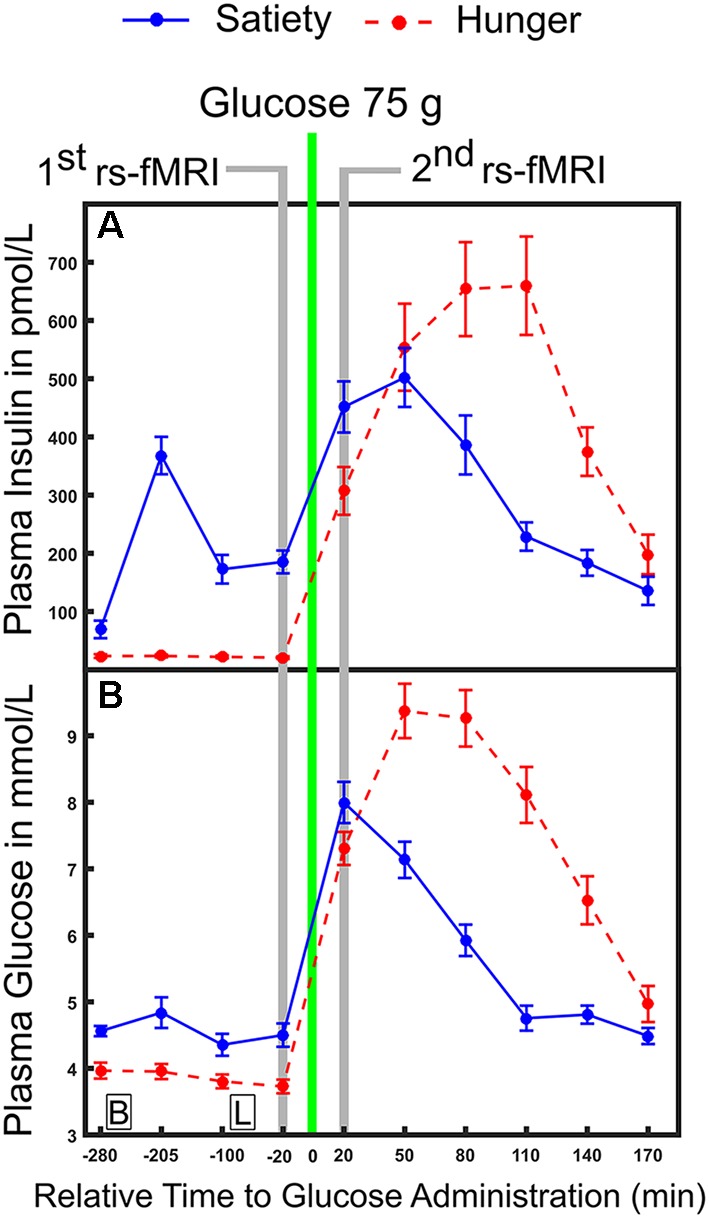
Time course of physiological parameters under hunger and satiety conditions. **(A)** Mean plasma concentration of insulin and **(B)** glucose under hunger and satiety conditions. Glucose was ingested at time point 0. The time-points of the first and second functional magnetic resonance imaging (fMRI) investigations are marked in gray. Boxes on the bottom of the graph indicate the time points of meals on the second day (B, breakfast; L, lunch at 09:00 am and 12:00 pm, respectively). The error bars represent the standard deviation.

In the hunger condition, subjects began fasting (no food or beverages, except water) from 11:00 pm the night before the examination started until the end of the second resting state recording. In the satiety condition, three standardized meals per day were provided. Standardized meals were served according to recommendations of the clinical diabetes counseling department at the University Medical Campus Schleswig-Holstein: Breakfast (25% protein, 50% carbohydrate and 25% fat), lunch (20% protein, 63% carbohydrate and 17% fat) and dinner (22% protein, 60% carbohydrate and 18% fat) were provided at 09:00 am, 12:00 pm and 07:00 pm, respectively. In both sessions, subjects arrived at the sleep lab at 08:00 am. Nurses inserted a cannula into a peripheral vein on the back of the hand to collect blood samples. The first blood sample for defining the basal blood glucose and insulin levels was taken at 08:45 am. Then the experimental protocol of the first day was completed and nine blood samples were collected across different time points. More details on the exact timing for obtaining blood samples can be found in our previous work (please see the “Experimental design” section and Figure 1 in Al-Zubaidi et al., 2018). All individuals stayed and slept overnight in the sleep laboratory. The next morning, blood samples were drawn at 08:45 am and at fixed time points throughout noon until the MRI sessions at 01:05 pm. For the resting state fMRI recording (duration 6 min), subjects were instructed to lie still inside the scanner with their eyes closed and to not engage in any particular cognitive activity. After the MRI measurements, subjects were brought back to the sleep lab and provided blood samples every 30 min until 04:45 pm on the same day. To investigate the changes in plasma glucose and insulin levels and to relate them to the fluctuation of resting brain activity, the four blood samples that were collected before i.e., 20, 100, 205 and 280 min before 75 (g oral dose of glucose) and the six blood samples that were taken after (20, 50, 80, 110, 140 and 170 min after glucose intake) oral glucose administration were used for the analyses (Figure 1). For each condition subjects rated how hungry they were 20 min before and 20 min after oral glucose administration and this was carried out using a visual analog scale from 0 (not hungry at all) to 9 (very hungry).

### Image Acquisition

We used a 3-T Philips Achieva scanner (Philips Medical Systems, Best, Netherlands) and a standard eight-channel phased array head coil to record functional and structural images. Functional images were acquired with a gradient-echo-EPI sequence in ascending interleaved fashion (repetition time TR = 2,000 ms; echo time TE = 28 ms; voxel dimensions 3 × 3 × 3 mm; field of view 192 × 192 mm^2^; 80° flip angle; 40 slices). 178 whole-brain volumes were recorded for each of the two runs per session. Further, the high-resolution structural T1 image consisted of 180 sagittal slices were acquired by applying a T1-weighted 3D turbo gradient-echo sequence with SENSE (image matrix 240 × 240; voxel dimensions 1 × 1 × 1 mm; field of view 240 × 240 mm^2^; 1 mm slice thickness; 9° flip angle).

### Handling and Analyses of Blood Samples, Hunger Ratings

Plasma glucose concentration was measured with B-Glucose-Data-Management system (HemoCue GmbH, Grossostheim, Germany). The blood samples were, if necessary, cooled and immediately centrifuged. The supernatants were stored at −80°C until analyses. Serum insulin concentration was measured by commercial enzyme-linked immunoassay (Immulite DPC, Los Angeles, USA; insulin: intra-assay coefficient of variation (CV) <1.5% and inter-assay CV <4.9%).

**Figure 2:**
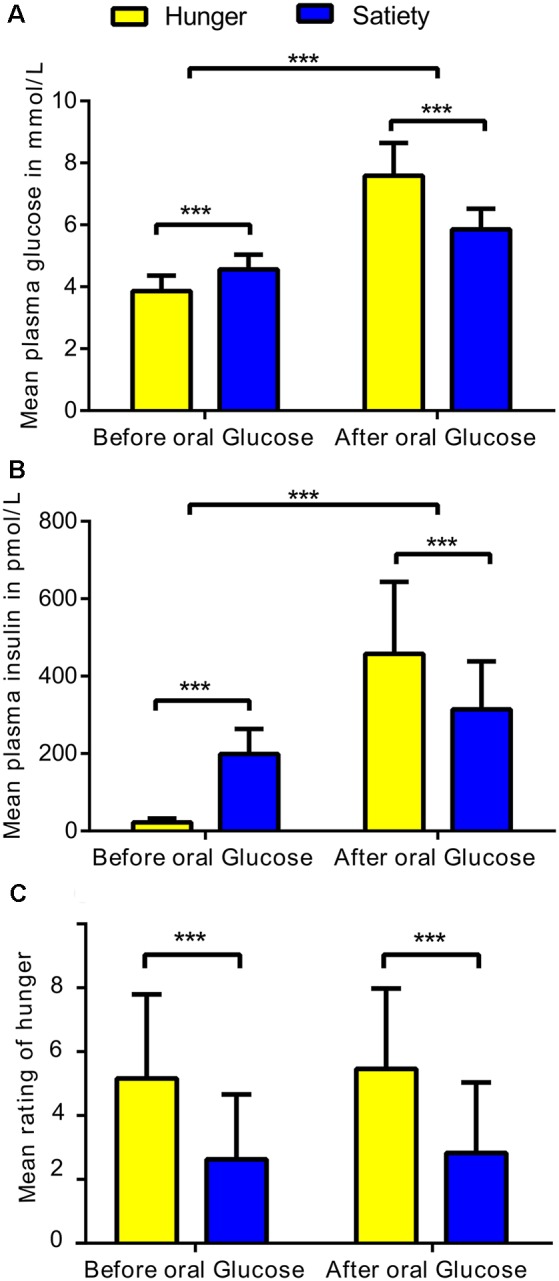
Statistical differences in physiological and behavioral parameters under hunger and satiety conditions. **(A)** Mean of plasma glucose, **(B)** plasma insulin, and **(C)** hunger ratings per factor: metabolic states (two levels: hunger and satiety) and oral glucose treatment (two levels: before, and after treatment) across participants. The error bars represent the standard deviation. ***Represent the significant differences (*p* < 0.0001).

The average plasma levels of glucose and insulin were calculated for each subject under hunger and satiety conditions before and after glucose treatment (Figure 2). To test the interaction between metabolic state (two levels: hunger, satiety) and the effect of glucose administration (two levels: before, and after treatment) on plasma glucose, plasma insulin, and subjective hunger rating, we applied a two-way repeated measures ANOVA for each of the dependent variables separately (SPSS, Version 22.0). Values are reported as mean (M) and standard deviation (± SD).

We also ran a Pearson correlation analysis to investigate the associations between changes of the subjective feeling of hunger with changes plasma glucose and insulin as well as with changes in brain activity (i.e., clusters that survived the cluster-significance test).

### Functional Image Preprocessing and Analyses

Part of preprocessing on functional images was initially carried out using FSLv5.0 (available at http://fsl.fmrib.ox.ac.uk/fsl/) to implement ICA-based strategy for automatic removal of motion artifacts (ICA-AROMA) for head motion correction (Pruim et al., 2015a). It has been shown that ICA-AROMA enhances the sensitivity and specificity of rs-fMRI activation and connectivity analyses (Pruim et al., 2015a). To improve inter-subject alignment (Klein et al., 2009; McLaren et al., 2010), the spatial preprocessing of the data were performed with the statistical parametric mapping 12b (SPM12b; available at http://www.fil.ion.ucl.ac.uk/spm/) in MATLAB (MathWorks, Natick, MA, USA) and data processing assistant for resting-state fMRI toolbox (DPARSF advanced edition, version 3.2, available at http://rfmri.org/DPARSF).

**Figure 3:**
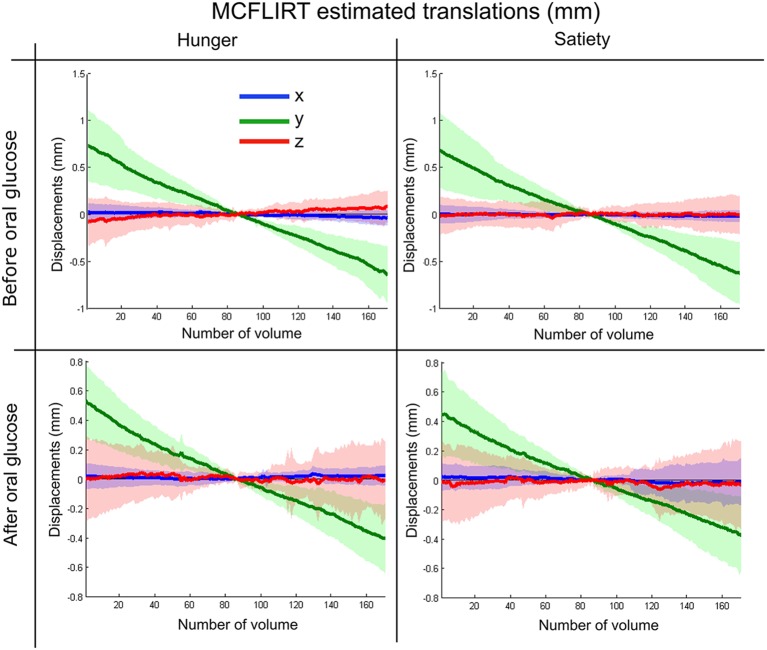
Summary of MCFLIRT estimated translation parameters for each experimental condition.

The rs-fMRI images were preprocessed as the following: (i) we discarded the first seven functional volumes from each participant’s four runs to allow steady-state tissue magnetization; (ii) we manually reoriented all functional volumes to the anterior commissure; (iii) we implemented head movement correction during data acquisition by volume-realignment to the middle volume using MCFLIRT (Jenkinson et al., 2002). The MCFLIRT results in estimated realignment parameters for each experimental condition were summarized in Figure 3 for motion translation and Supplementary Figure S1 for motion rotation parameters; (iv) we applied ICA-AROMA to the data in order to identify and remove motion-related components using four spatial and temporal features as summarized in Al-Zubaidi et al. (2018) and detailed in Pruim et al. (2015b). Then the denoised functional and structural images were further preprocessed with DPARSF toolbox as following: (v) we co-registered the T1 structural image to the mean functional image; (vi) we ran a segmentation protocol to distinguish gray matter, white matter and cerebrospinal fluid (CSF); (vii) we applied bias correction and spatial normalization of the T1 structural image and adjusted them to the Montreal Neurological Institute (MNI) template using diffeomorphic anatomical registration through exponentiated Liealgebra (DARTEL) algorithm (Ashburner and Friston, 2005); (viii) we performed nuisance regression (including white matter and CSF signals) to reduce the influence of unspecific physiological effects on BOLD signal (Liu, 2016); (ix) we spatially normalized functional images to the MNI-template using the normalization parameters estimated by DARTEL algorithm with a 3 mm isotropic voxels size; (x) we performed spatial smoothing with a 6 mm full width at half maximum (FWHM) Gaussian kernel; and (xi) finally, we masked all functional images with a gray matter mask, which was calculated by averaging the gray matter masks of all subjects (Al-Zubaidi et al., 2018).

We carried out a first-level analysis on each subject and each run by calculating the fALFF on the preprocessed data using DPARSF toolbox (Yan and Zang, 2010). DPARSF has built-in Fast Fourier Transform (FFT) to convert time series for each voxel to the frequency domain and compute the power spectrum. This procedure estimates the amplitude of each frequency as the square root of the power spectrum. Last, the total amplitude of the low-frequency range (0.01–0.08 Hz) was divided by that of the entire frequency range 0–0.25 Hz (TR = 2 s). Put simply, fALFF is the ratio of rs-fMRI signal fluctuation in the low-frequency range relative to the entire frequency range (Zou et al., 2008). Individual fALFF maps were transformed to z-scores to reduce the effect of individual variability (Al-Zubaidi et al., 2018).

### Statistical Analysis of Functional Images

To capture the modulatory impact of glucose administration on the association between the activity of brain regions and plasma insulin or glucose levels under different metabolic states, we performed two multiple linear regression analyses (MLRA) using SPM12, the first “before” and the second “after” oral glucose treatment. Each MLRA was designed with two covariates (plasma insulin and glucose levels). Briefly, MLRA is used to describe how a “linear combination” of multiple variables, called independent or explanatory variables, to predict a single response variable, referred to as the dependent or target variable. The contribution of each independent variable to the model is assessed by looking at the regression coefficients (Nathans et al., 2012). In this study, we used MLRA to figure out the contribution of plasma insulin (an independent variable) to the prediction of brain activity (the dependent variable) when taking the effect of plasma glucose (another independent variable) into account (Kiebel and Holmes, 2007; Field, 2014), and *vice versa*. Accordingly, our multiple linear regression model is:

(1) $$Y_{j}=\mu +x_{j1}\beta_{1}+x_{j2}\beta_{2}+\in_{j},$$

where *Y*_j_ is a dependent variable (such as fALFF at a particular voxel) and *j* = 1,…,*J* indexes the observation. The regression coefficient *μ* represents a constant term (the mean of the dependent variable when all predictors are zero), while *β*_1_ represents the regression slope, which quantifies the association of *Y* with *x*_1_ (such as plasma insulin), adjusting for the effect of *x*_2_ (plasma glucose) on *Y* and vice versa for *β*_2_ and ∈ is the error associated with the regression (the variance of the dependent variable from its mean when all predictors are zero). The parameters were estimated by using the least squares method. To find significant voxels whose activity was affected by hunger vs. satiety or by glucose or insulin, we used one-sample *t*-tests for each regression coefficient on the voxel level per MLRA (see section below). The resulting statistics indicate whether the regression coefficient of a particular voxel is significantly different from the error in that estimate (Field, 2014). To correct for multiple comparisons, the topological false discovery rate (FDR) *q* = 0.05 was employed with a cluster defining threshold of *p* < 0.001 for the *t*-tests (Chumbley et al., 2010).

**Figure 4:**
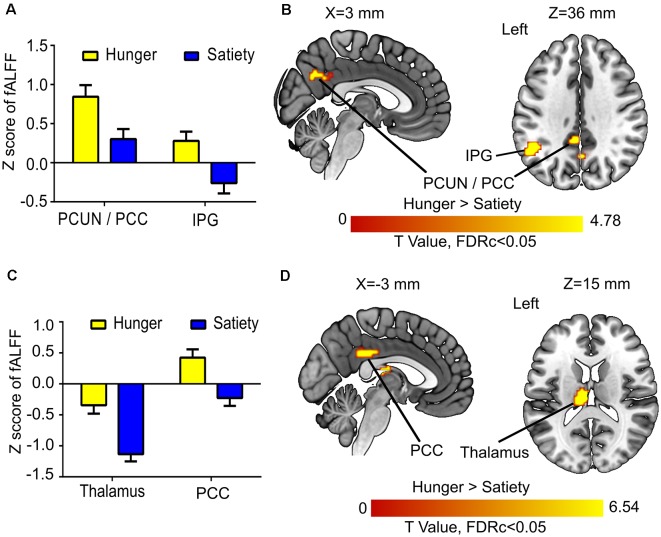
Hunger vs. satiety effects on brain activity. **(A)** Mean fractional amplitude of low-frequency fluctuation (fALFF) value of all voxels of a significant cluster per condition across participants. **(B)** Results of the first model (before glucose administration). **(C)** Mean fALFF value of all voxels of a significant cluster per condition across participants. **(D)** Results of the second model (after glucose administration). Abbreviations: PCC, posterior cingulate cortex; PCUN, precuneus; IPG, inferior parietal gyrus.

In the 1st MLRA (before glucose administration), we contrasted hunger vs. satiety states while controlling the moderating fluctuation of glucose and insulin. We calculated differences [delta (Δ) = hunger-satiety] of fALFF maps before oral glucose treatment. For glucose and insulin, the area under the concentration-time curve (AUC) of the first four samples (Figures 1A,B) was calculated and the Δ of the AUC was obtained. The AUC has been used as an index to assess the regulation of glucose and insulin (Tzagournis and Skillman, 1970; Owen et al., 2012). It was calculated using the standard trapezoid method, which is computing the AUC with respect to ground (see formula 2 in Pruessner et al., 2003). The group level analysis was performed using Δ AUC of glucose and insulin as covariates (independent variables) and the Δ fALFF maps as the dependent variable in the MLRA. Notably, the Δ AUC of glucose and insulin were not significantly correlated (|*r*| = 0.3, *p* = 0.1). To check whether the AUC of plasma glucose and insulin influence our findings, we also recomputed the 1st MLRA by including the Δ plasma glucose and insulin based on the value 20 min before glucose ingestion (Figure 1) as independent variables. The results of this model (see Supplementary Table S1 and Supplementary Figure S2 in the supplementary material) were similar to the results of the model with AUC (see Table 1 and Figure 4A). The AUC provides an overview of plasma glucose and insulin level profiles under diet or standard meal vs. time (Johnson et al., 2006). Also, we believe that the changes in brain activity before glucose ingestion may be related to profile change more than single glucose and insulin values. Therefore, we will report the AUC model results only.

**Table 1:** Changes and associations of fractional amplitude of low-frequency fluctuation (fALFF) with food conditions and hormone levels.

Regions	Hes.		K		Local maxima (mm)		
					*x*	*y*	*z*
**Before glucose administration: hunger > satiety**							
Inferior parietal gyrus	L	0.03	24	4.78	−45	−51	36
Inferior parietal gyrus	L			4.51	−51	−57	36
Posterior cingulate cortex	L	0.01	34	4.70	−6	−45	36
Posterior cingulate cortex	L			4.16	0	−48	30
Precuneus	R			4.04	9	−54	30
**After glucose administration: hunger > satiety**							
Thalamus	L	0.001	55	6.54	−3	−21	15
Thalamus	L			5.18	−12	−21	15
Thalamus	L			4.86	−9	−33	5
Posterior cingulate cortex	L	0.002	45	5.68	0	−33	33
Posterior cingulate cortex	L			4.42	−9	−33	33
**Correlations between changes in physiological and neural of metabolic states (hunger-satiety), after glucose administration**							
**Positive correlation with plasma glucose levels**							
Fusiform gyrus	L	0.04	25	4.07	−42	−63	−12
Fusiform gyrus	L			4.05	−27	−72	−15
Fusiform gyrus	L			3.39	−33	−66	−15
**Negative correlation with plasma insulin levels**							
superior frontal gyrus	L	0.04	19	5.50	−12	21	63
superior frontal gyrus	L			4.47	−15	15	57
superior frontal gyrus	L			3.99	−6	33	57
Posterior insula	L	0.004	19	4.39	−33	−9	9
Posterior insula	L			4.31	−36	−21	9

*Notes: The table shows three local maxima [Montreal Neurological Institute (MNI) coordinates] more than 8.0 mm, the adjusted (adj.) p-values are reported at *p* < 0.001 (height threshold) and *q* < 0.05 (FDR extent threshold). T, peak of T values; K, cluster size; Hes, hemisphere; L, left; R, right*.

The 2nd MLRA (after glucose administration) had a similar design as first MLRA. Differences (Δ) of amplitude rs-fMRI signals were calculated by subtracting the fALFF maps of hunger condition from satiety condition. The Δ calculated for glucose and insulin was based on the value 20 min after glucose ingestion (Figure 1) only to keep the temporal association of endocrine and functional imaging data as clear as possible. Δ glucose and Δ insulin were used as covariates in the MLRA. Again, glucose and insulin were not significantly correlated (|*r*| = 0.14, *p* = 0.5). Additional glucose and insulin samples collected after oral glucose intake were not included in this analysis, because they were taken after the second rs-fMRI recording.

To ensure that the correlation between specific brain regions and glucose or insulin values is not biased (Esterman et al., 2010), we extracted fALFF values by averaging across voxels in each cluster that survived the cluster-significance test. Then, we performed the leave-one-out cross-validation (LOOCV) of Pearson correlation to evaluate the relationship between averaged ΔfALFF values of each brain region with Δ of the plasma glucose and insulin. Finally, we performed full and partial correlation analyses between clusters linked to plasma insulin levels to investigate the association between them and the effects of glucose and insulin values on that association.

Finally, to investigate the acute effect of glucose administration on the interaction between brain activity and physiological changes, we performed two additional MLRA. The 3rd and 4th MLRAs estimate the correlations between changes in fALFF (Δ fALFF = pre-post glucose ingestion) and changes in plasma glucose/insulin (Δ of plasma glucose/insulin were calculated based on the value of 20 min before and after glucose ingestion; Figure 1) under satiety and hunger conditions separately, respectively.

We used the automated anatomical labeling (AAL) atlas (Tzourio-Mazoyer et al., 2002) included in the xjView toolbox^1^ to label the anatomical location of significant clusters. Thalamic nuclei were identified by applying the MNI-based Morel Atlas (Jakab et al., 2012).

## Results

### Physiological and Behavioral Effects

The ANOVA of plasma glucose concentration (Figure 2A) revealed a significant metabolic state (hunger/satiety) and glucose administration interaction (*F*_(1,23)_ = 53.6, *p* < 0.001). The main effects metabolic state (*F*_(1,23)_ = 23.1, *p* < 0.001) and glucose treatment (*F*_(1,23)_ = 256, *p* < 0.001) were significant as well. The interaction was driven by an increased area under curve for glucose following the exogenous glucose challenge in the hunger (M = 7.6 mmol/l, SD = 1.1) compared to the satiety condition (M = 5.9 mmol/l, SD = 0.6) whereas this was reversed before glucose treatment with higher plasma glucose level in the satiety (M = 4.6 mmol/L, SD = 0.5) compared to the hunger condition (M = 3.9 mmol/L, SD = 0.5).

For insulin, a significant metabolic state and glucose treatment interaction was found (*F*_(1,23)_ = 101.8, *p* < 0.0001). In addition, a significant main effect glucose treatment (*F*_(1,23)_ = 106, *p* < 0.001) was revealed, but the main effect metabolic state was not significant (*F*_(1,23)_ = 0.65, *p* = 0.4). As shown in Figure 2B, before glucose treatment the plasma insulin level for the satiated state (M = 198.8 pmol/L, SD = 65.2) was higher compared to the hunger state (M = 22.5 pmol/L, SD = 10.1). In contrast, after glucose treatment, the plasma insulin level was greater in the hunger (M = 457.9 pmol/L, SD = 185.6) compared to the satiated state (M = 314.6 pmol/L, SD = 123.5), indicating reduced responsiveness to circulating insulin.

The analysis of the subjective hunger ratings revealed higher hunger ratings in the fasting condition compared to satiety (*F*_(1,23)_ = 28.9, *p* < 0.001), confirming the success of our fasting treatment (Figure 2C). There was neither a main effect of glucose treatment and nor an interaction between both factors. In addition, we found no significant correlations between subjective feeling of hunger with changes plasma glucose and insulin as well as with changes brain activity (i.e., clusters that survived the cluster-significance test). These results are shown in Supplementary Figure S3.

### Resting State fMRI: Hunger vs. Satiety Effects

The 1st MLRA (before glucose administration) revealed differences of resting brain activity as a function of hunger in the PCC and the left anterior precuneus (PCUN), as well as in the left inferior parietal gyrus (IPG; Figures 4A,B, Table 1). The 2nd MLRA (after glucose administration) showed an increase of the fALFF signal in the hunger compared to the satiety condition in the left thalamus and the left PCC (Figures 4C,D, Table 1).

### Resting State fMRI: Before vs. After Glucose Treatment

**Figure 5:**
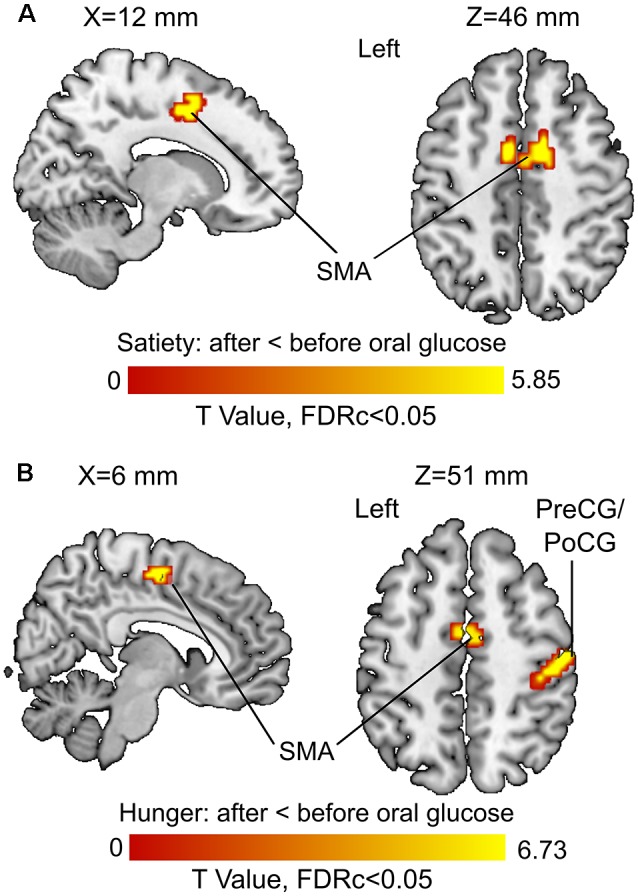
Before vs. after glucose administration on brain activity. **(A)** Results of the third model (under satiety condition). **(B)** Results of the fourth model (under hunger condition). Abbreviations: SMA, supplementary motor area; PreCG, precentral gyrus; PoCG, postcentral gyrus.

The 3rd (under satiety condition) and 4th (under hunger condition) MLRA results showed that the resting brain activity of supplementary motor area (SMA) was significantly decreased after glucose ingestion (Figures 5A,B, Table 2). In the hunger condition (4th MLRA), a reduced resting brain activity was evident in precentral gyrus (PreCG) and the postcentral gyrus (PoCG) after glucose administration (Figure 5B, Table 2).

**Table 2:** Influences of oral glucose administration on brain activity under hunger and satiety conditions.

Regions	Hes.	*p*(adj.)	K	T-value	Local maxima (mm)		
					*x*	*y*	*z*
**Satiety condition: after oral glucose < before oral glucose**							
Supplementary motor area	L	0.003	39	5.85	−3	−18	54
Supplementary motor area	R	<0.001	86	4.77	12	3	45
Supplementary motor area	L			4.63	−6	3	48
**Hunger condition: after oral glucose < before oral glucose**							
Postcentral gyrus	R	<0.001	136	6.73	51	−18	51
Precentral gyrus	R			5.61	30	−6	69
Precentral gyrus	R			5.49	36	−15	66
Precentral gyrus	L	<0.001	51	6.60	−24	−12	69
Supplementary motor area	L	<0.001	58	5.52	−3	−6	54
Supplementary motor area	R			4.88	6	−15	54

*Notes: The table shows three local maxima (MNI coordinates) more than 8.0 mm, the adjusted (adj.) p-values are reported at *p* < 0.001 (height threshold) and *q* < 0.05 (FDR extent threshold). T, peak of T values; K, cluster size; Hes, hemisphere; L, left; R, right*.

### Correlations Between Physiological and Neural Effects

For the 1st, 3rd and 4th MLRAs, we found no correlation between changes in resting brain activity and changes in glucose and insulin passing the correction for multiple comparisons.

**Figure 6:**
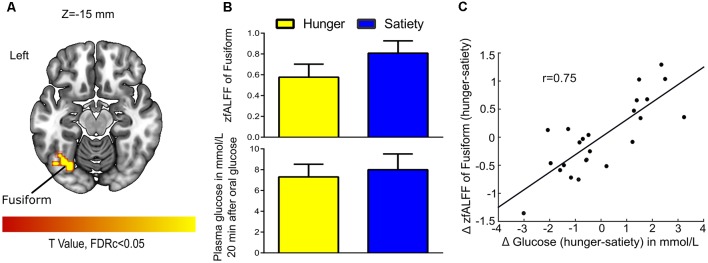
Glucose-associated low-frequency BOLD fluctuations. **(A)** The Δ (hunger-satiety) of fALFF in Fusiform connected with Δ glucose plasma after glucose administration. The statistical image was assessed for cluster-wise significance using a cluster defining threshold *P* < 0.001, 0.05 false discovery rate (FDR) corrected at the cluster level. **(B)** Amplitude of BOLD signal in the fusiform gyrus and plasma glucose levels in the hunger and satiety states. **(C)** The LOOCV showed that a positive correlation (*r* = 0.75) between the average Δ fALFF value of fusiform cluster and Δ plasma glucose, adjusted for Δ plasma insulin level. LOOCV, leave-one-out cross-validation.

For 2nd MLRA (i.e., hunger-satiety), after glucose administration and while controlling for effects of Δ insulin we found a correlation between Δ fALFF and Δ plasma glucose in the left fusiform gyrus (Figures 6A,B, Table 1). A LOOCV between the average Δ fALFF values of the fusiform cluster and Δ plasma glucose after data being adjusted for Δ plasma insulin level (Figure 6C) revealed a significant positive correlation (*r* = 0.75).

**Figure 7:**
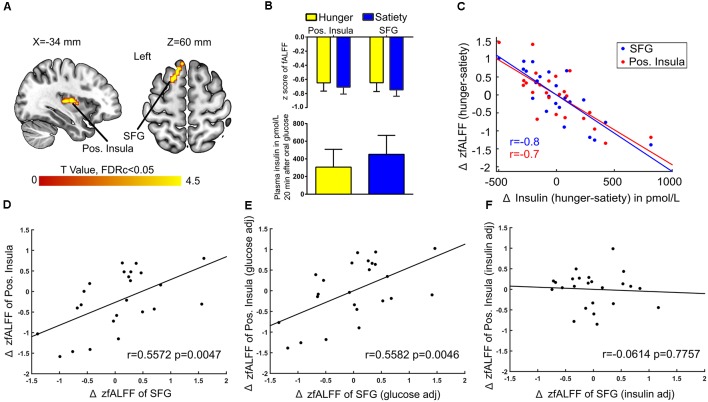
Insulin-associated low-frequency BOLD fluctuations. **(A)** The Δ (hunger-satiety) of fALFF in the left posterior (pos.) insula and superior frontal gyrus (SFG) connected with Δ plasma insulin after glucose administration. The results were assessed for cluster-wise significance using a cluster defining threshold *P* < 0.001, 0.05 FDR corrected at the cluster level. **(B)** SFG and pos. insula amplitude values of the BOLD signal and plasma insulin levels in the hunger and satiety states. **(C)** Scatter plot of the correlation between Δ plasma insulin and average Δ fALFF value of left pos. Insula (LOOCV of *r* = 0.7; red dots and line) and left SFG (LOOCV of *r* = 0.8; blue dots and line), adjusted for Δ plasma glucose level. Panels **(D,E)** represent a significant positive correlation between the average Δ fALFF values of left pos. insula and left SFG when the data was either not adjusted or adjusted for Δ plasma glucose, respectively. **(F)** The correlation between the average Δ fALFF value of left pos. insula and left SFG was no longer significant when the data was adjusted for Δ plasma insulin level, which suggests the effects were driven by plasma insulin. LOOCV, leave-one-out cross-validation.

Next, when controlling for Δ plasma glucose we found a connection between Δ fALFF and Δ plasma insulin after glucose administration in the left posterior insula and SFG (Figures 7A,B). A significant negative correlation (*r* = −0.7) was found between average Δ fALFF of the posterior Insula cluster and Δ plasma insulin (Figure 7C; red dots and line), as well as a significant negative correlation (*r* = −0.8) between average Δ fALFF SFG and Δ plasma insulin (Figure 7C; blue dots and line).

A potential influence of plasma insulin and plasma glucose on the association between Δ fALFF posterior insula and Δ fALFF SFG was tested by using the average Δ fALFF in these clusters and revealed a significant positive correlation (*r* = 0.5, *p* = 0.004, Figure 7D) which survived when adjusting for Δ plasma glucose(*r* = 0.5, *p* = 0.004, Figure 7E) but not when adjusting for Δ plasma insulin level (*r* = −0.06, *p* = 0.7, Figure 7F).

## Discussion

In the present study, we investigated the interaction of hunger and satiety with plasma glucose and plasma insulin levels before and after glucose administration and explored this interaction’s impact on changes in resting state fMRI. We used fALFF, a measure reflecting the temporal fluctuation of the BOLD signal, to study neural activity and connectivity. Multiple linear regressions analyses (MLRA) with two covariates (glucose, insulin) was used to assess the moderating influence of these covariates on the differences between hunger and satiety conditions.

Expectedly, plasma glucose and insulin levels differed between hunger and satiety conditions and were also differentially influenced by glucose administration (Figures 1, 2). In the satiety condition, a major peak in insulin and glucose levels around 3 h before glucose ingestion is related to the breakfast consumed in this condition. Generally, before glucose administration, insulin and glucose levels are higher in the satiety compared to fasting condition, which is the expected result (Johnson et al., 2006). After ingestion of glucose, there is a massive rise of both insulin and glucose levels as expected. This curve is higher and wider for both parameters in the fasting condition, which can be explained by post-fasting glucose intolerance (Johnson et al., 2006). Furthermore, the delay in the time till the maximum is reached in the fasting condition compared to the satiety condition can be explained with a delayed gastric emptying time for fasted individuals (Corvilain et al., 1995). The analyses of the fALFF data revealed an increased BOLD signal amplitude in the PCC in hunger relative to satiety that was independent of glucose and insulin plasma levels. Brain activity reflecting the difference between hunger vs. satiety was modulated by changes in glucose and insulin plasma levels in fusiform and PINS after oral glucose intake, respectively.

### Physiological and Behavioral Effects

The results for glucose (Figure 2) suggest that hunger is stimulated, at least in part, by changing the blood glucose level (Pannacciulli et al., 2007). Also, a decrease in insulin concentrations in fasting compared to the fed state might function as a trigger to increase food intake (i.e., carbohydrate intake) in lean men (Mars et al., 2005). In addition, a meta-analysis showed that high insulin levels in response to a meal suppress appetite (Flint et al., 2007). Therefore, glucose and insulin may act as biomarkers of the satiety (de Graaf et al., 2004). Hunger ratings have been shown to increase with blood glucose concentration of 4.4 mmol/L (85 mg/dL) or lower (Ciampolini and Bianchi, 2006). In our study, the mean blood glucose level was 3.6 mmol/L under hunger condition (Figure 1B), and the feeling of hunger was higher under hunger relative to satiety condition before the fMRI scans (Figure 2C). These findings suggest that the experimental manipulation worked.

### Resting-State fMRI: Hunger vs. Satiety Effects

The amplitude of the rs-fMRI signal was increased for hunger vs. satiety in the left IPG (IPG) before oral glucose treatment and in the PCC before and after oral glucose treatment (Figure 4). Several studies using visual stimuli showed enhanced activity of IPG, PCC, precuneus and insula during food choice vs. non-food choice under hunger conditions (Führer et al., 2008; Charbonnier et al., 2015). Moreover, PCC and precuneus are core hubs of the DMN (Cavanna and Trimble, 2006; Benedict et al., 2008). The DMN is activated during the rs-fMRI paradigm and deactivated during task-based fMRI, when attention is oriented towards internal rather than external stimuli (Gerozissis, 2003; Buckner et al., 2008). In line with that, the DMN, especially in the PCC, has been shown to be involved in self-referential processes (Buckner and Carroll, 2007). A study using ICA approach to investigate the FC of obese/overweight subjects found a reduction of DMN activity associated with a decrease in hunger ratings and fat mass after 6 months of exercise (McFadden et al., 2013). In the current experiment, the increased fALFF in PCC region seems to be driven by metabolic state (hunger vs. satiety; Figure 4) independently of changes in the plasma glucose and insulin levels. Consistent with our results, DMN activity has been shown to be positively correlated with lower blood glucose levels (Bolo et al., 2015) and with hunger ratings (McFadden et al., 2013). Our findings in the current and previous study (Al-Zubaidi et al., 2018) about increased activation of the DMN (i.e., PCC) under hunger condition might be indicative of increased self-focus during food deprivation.

We found a significant increase of fALFF in the left mediodorsal thalamus under hunger conditions and a decrease in satiety after (but not before) glucose treatment indicating an interaction between metabolic state and glucose administration in this region. Thalamic brain activity has been reported to vary as a function of hunger (Tataranni et al., 1999; Zhao et al., 2016), ghrelin application (Higgins et al., 2007) and glucose infusion (Jones et al., 2012; Little et al., 2014). A previous study (Kroemer et al., 2013a) reported a positive correlation between fasting levels of ghrelin and thalamic reactivity to food images, though this effect was not further modulated by glucose intake (75 g glucose). The thalamus is central for the integration of proprioceptive information stemming from the gastrointestinal (GI) tract (Kelley et al., 2005; Little et al., 2014). Specifically, afferent information from this tract reaches the thalamus *via* the vagus nerve (Coss-Adame and Rao, 2014). Consistent with this observation, Rolls (2005, 2006) postulated that the mediodorsal thalamus impacts short-term eating behavior. Our results agree with these proposals in that mediodorsal thalamus activity is partly dependent on metabolic state and associated with glucose metabolism.

### Resting State fMRI: Before vs. After Glucose Treatment

FALFF was lower in the left SMA after glucose ingestion (Figure 5). The SMA is crucial for planning movements (Cunnington et al., 2002; Nachev et al., 2008) and observation of actions (Grèzes and Decety, 2002). The response to glucose ingestion in the form of lower activity in the SMA could potentially lead to suppressed action planning or initiation because of an alteration in the available energy resources of the body. Therefore, plasma glucose might have an effect on brain regions controlling attention, food evaluation and motor planning. Further research is needed to back up this interpretation in the form of task-related fMRI or behavioral studies that show such functional differences in dependency of blood glucose levels.

### Correlations Between Physiological and Neural Effects

We observed that changes in plasma insulin levels (hunger vs. satiety) after oral glucose administration were negatively associated with changes in the amplitude of the BOLD signal in the left PINS and the left SFG). Furthermore, we found a significant positive correlation between left PINS and left SFG when the ΔfALFF values were adjusted for the Δplasma glucose, while this relationship disappeared when the ΔfALFF values were adjusted for the Δplasma insulin (Figure 7). In contrast, changes in plasma glucose levels were positively correlated with changes in the fALFF in the left fusiform after oral glucose administration (Figure 6). The PINS is involved in sensory, motor and interoception taste intensity (Kurth et al., 2010; Kelly et al., 2012; Nieuwenhuys, 2012; Tang et al., 2012) but is also activated during hunger (Tataranni et al., 1999), during food craving (Siep et al., 2012), and on receiving an appetitive drink (Bohon and Stice, 2011). The SFG is activated in response to appetitive stimuli in fasted subjects (Malik et al., 2011; Martens et al., 2013), and it is frequently involved in inhibiting approach behavior towards food (Gautier et al., 2000; McCaffery et al., 2009; Batterink et al., 2010). The fusiform gyrus harbors high-level visual processes (e.g., face recognition; Hadjikhani et al., 2004), but has been shown to be activated in response of food cues after glucose administration (Heni et al., 2014), to high vs. low caloric food pictures in lean subjects (Kroemer et al., 2013b), and as a function of increasing hunger (Zhao et al., 2016).

Our data showed that oral glucose administration modulates the insulin-dependent association between PINS and SFG, as well as the association between left fusiform gyrus activity with plasma glucose. Our results suggest that the fusiform, PINS, and SFG activity is modulated by an intricate interplay of glucose and insulin levels, most likely to regulate ingestive behavior by differential engagement of attentional, executive and effective processes.

These findings dovetail nicely with results from rs-fMRI studies demonstrating that body weight and insulin levels influence the FC within and between cognitive and homeostatic brain regions (Kullmann et al., 2012, 2013).

### Limitations

Although our study was tightly controlled, there are some limiting factors that should be taken into account. First, although the hypothalamus is a central region for the regulation of energy homeostasis (Reno et al., 2016; Rosario et al., 2016), it was not modulated in the current study, which may be due to insufficient resolution of this small structure in fMRI imaging (De Silva et al., 2012) and the use of cluster-level correction (number of voxels per cluster; Woo et al., 2014). Specific scanning protocols targeted at subcortical structures in combination with region-of-interest analyses might be needed to detect hypothalamic effects. Second, we only investigated healthy young male participants. Therefore, our results may not be readily generalizable to other populations. Second, we only investigated healthy young male participants. Therefore, our results may not be readily generalizable to other populations. For instance, Haase et al. (2011) reported gender differences in response to sweet taste under hunger and satiety conditions in several brain regions. Also, the effects of insulin signaling on the human brain show sex differences (Benedict et al., 2008; Ghasemi et al., 2013). There have been multiple previous studies addressing differences in brain activity between lean and obese subjects (Hogenkamp et al., 2016). It is thus conceivable that the rs-fMRI measures used in the current study might also differ between lean and obese subjects. Furthermore, the correlation analysis after glucose administration was only performed with endocrine data narrowly associated with the fMRI data collection to keep this connection as clear as possible. Finally, our study focused on revealing correlations between plasma insulin and glucose concentrations and brain activity under different metabolic states. To extend these results, future studies might employ a longitudinal design with multiple fMRI sessions during the course of the fasting and control conditions and collection of the endocrine data. If the shown correlations persist or build up in a sensible way over time these results would be a strong addition to the correlational findings of this study. As this study was designed to confirm previous findings and to show the validity of these potential correlations in a physiological setting with an oral glucose intake intervention we only performed two fMRI sessions. To establish a causal relationship in contrast to our correlational findings, an experimental manipulation using insulin clamp and/or glucose clamp techniques is needed.

## Conclusion

Our results suggest that plasma glucose and insulin respond similarly to oral glucose intake depending on metabolic states (hunger vs. satiety) and that these responses are related to different neural processing in the brain. Changes in plasma glucose were associated with changes of activation patterns in the fusiform gyrus, while changes in plasma insulin enhanced connectivity between the posterior insula and SFG when added as a covariate in the MLRA, indicating that changes in plasma insulin levels were at least partially responsible for the augmented connectivity. This connectivity appears to be related to alleviating an acute hemostatic energy deficit. These results contribute to identifying the neural mechanisms through which insulin regulates food intake (Kullmann et al., 2017). All in all, our findings expand existing neural models of homeostatic energy and highlight the complex nature of food intake and hormone-relationships in humans.

## Ethics Statement

The study was approved by the ethics committee of the University of Lübeck and carried out under the Declaration of Helsinki (2000). Before participation, each participant gave written informed consent.

## Author Contributions

KJ-C and TM designed the study and wrote the protocol. AA-Z, MH, JN and KJ-C participated in the data collection. AA-Z managed the literature searches and wrote the first draft of the manuscript. AA-Z and AM performed data processing and statistical analyses. MH, KJ-C, GB and TM helped with interpretation of data. All authors contributed to and had approved the final manuscript.

## Conflict of Interest Statement

The authors declare that the research was conducted in the absence of any commercial or financial relationships that could be construed as a potential conflict of interest.

## Footnotes

^1^http://www.alivelearn.net/xjview8

## Abbreviations

APCUNS: anterior precuneus
AUC: area under the concentration-time curve
BMI: body mass index
BOLD: Blood-oxygen-level-dependent
CSF: cerebrospinal fluid
DARTEL: diffeomorphic anatomical registration through exponentiated Liealgebra
CBF: cerebral blood flow
CNS: central nervous system
Δ: delta = hunger-satiety
DMN: default mode network
DPARSFA: data processing assistant for resting-state fMRI advanced edition
EPI: echo-planar imaging
fALFF: fractional amplitude of low-frequency fluctuations
FC: functional connectivity
fMRI: functional magnetic resonance imaging
FSL: FMRIB Software Library
FDR: false discovery rate
ICA-AROMA: ICA-based strategy for automatic removal of motion artifacts
IPG: inferior parietal gyrus
K: number of voxel per cluster
LOOCV: leave-one-out cross validation
M: mean
MLRA: multiple linear regression analysis
MNI: Montreal Neurological Institute
PCC: posterior cingulate cortex
PET: positron emission tomography
PINS: posterior insula
rs-fMRI: resting-state functional magnetic resonance imaging
rm-ANOVA: repeated measures analysis of variance
SD: standard deviation
SPM: statistical parametric mapping
SFG: superior frontal gyrus
TE: echo time
TR: repetition time.
