# Supplementary material for: Impact of Hunger, Satiety, and Oral Glucose on the Association Between Insulin and Resting-State Human Brain Activity
Source: Front Hum Neurosci. 2019 May 14;13:162. doi: 10.3389/fnhum.2019.00162 (PMC6544009; doi:10.3389/fnhum.2019.00162)
Supplement: Supplementary file 1 [file Data_Sheet_1.docx]

**Supplementary materials**

**Impact of hunger, satiety, and oral glucose on the association between insulin and resting-state human brain activity**

Arkan Al-Zubaidi^1^, Marcus Heldmann^1, 2^, Alfred Mertins^3^, Georg Brabant^4^, Janis Marc Nolde^1^, Kamila Jauch-Chara^5^, Thomas F. Münte^1, 2,*^

^1^ Dept. of Neurology, University of Lübeck, Lübeck, Germany

^2^ Institute of Psychology II, University of Lübeck, Lübeck, Germany

^3^ Institute for Signal Processing, University of Lübeck, Lübeck, Germany

^4^ Dept. of Internal Medicine I, University of Lübeck, Lübeck, Germany

^5^ Dept. of Psychiatry and Psychotherapy, Christian-Albrechts-University, Kiel, Germany

| 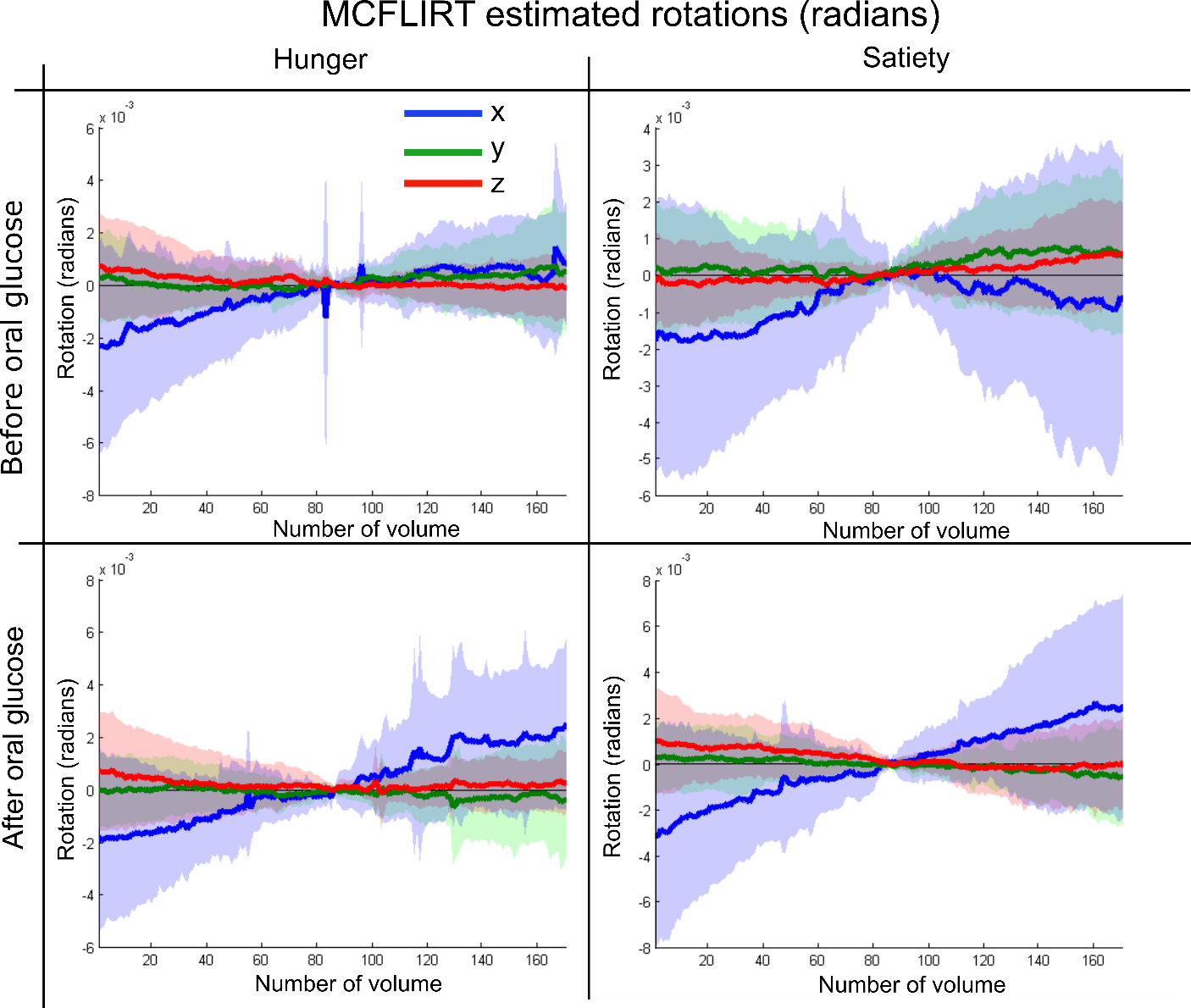 |
| --- |
| Figure S1. Summary of MCFLIRT estimated rotation parameters for each experimental condition. |

| 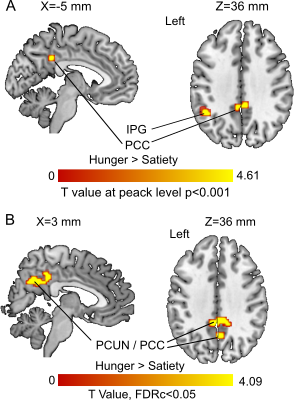 |
| --- |
| Figure S2. Reanalysis of the 1^st^ MLRA with single glucose and insulin values instead of AUC values. (A) The results under p(peak) levels and K>10. (B) The p-values are reported at p<0.005 (height threshold) and q<0.05 (FDR extent threshold). This figure is similar to Figure 4 in the manuscript. |

| Table S1. Reanalysis of the 1^st^ MLRA with single glucose and insulin values instead of AUC values. These results are similar to the results in Table 1 (in the manuscript) | | | | | | | | | | | | | | |
| --- | --- | --- | --- | --- | --- | --- | --- | --- | --- | --- | --- | --- | --- | --- |
| Regions | Hes. | | **p(peak)** | | | K | | T value | | Local maxima (*x y z*) (mm) | | | | |
| **Before glucose administration: hunger > satiety** | | | | | | | | | | | | | | |
| Inferior parietal gyrus | L | | <0.001 | | | 14 | | 4.61 | | -45 | | -51 | | 36 |
| Posterior cingulate cortex | R | | <0.001 | | | 11 | | 4.09 | | 6 | | -42 | | 36 |
| Posterior cingulate cortex | L | | <0.001 | | |  | | 3.37 | | -6 | | -45 | | 36 |
|  | | | | | | | | | | | | | | |
|  |  | | | **p(adj.)** |  | |  | |  | | | | | |
| **Before glucose administration: hunger > satiety** | | | | | | | | | | | | | | |
| Posterior cingulate cortex | | R | | 0.003 | 112 | | 4.09 | | 6 | | -42 | | 36 | |
| Posterior cingulate cortex | | L | |  |  | | 4.08 | | 0 | | -42 | | 42 | |
| Precuneus | | R | |  |  | | 4.04 | | 9 | | -54 | | 30 | |
| Notes: The table shows three local maxima (MNI coordinates) more than 8.0 mm, p(peak)-values are reported at peak level, p<0.001 and K>10. The adjusted (adj.) p-values are reported at p<0.005 (height threshold) and q<0.05 (FDR extent threshold). T=peak of T values. K=cluster size.  Hes. =hemisphere. L=left. R=right | | | | | | | | | | | | | | |

| 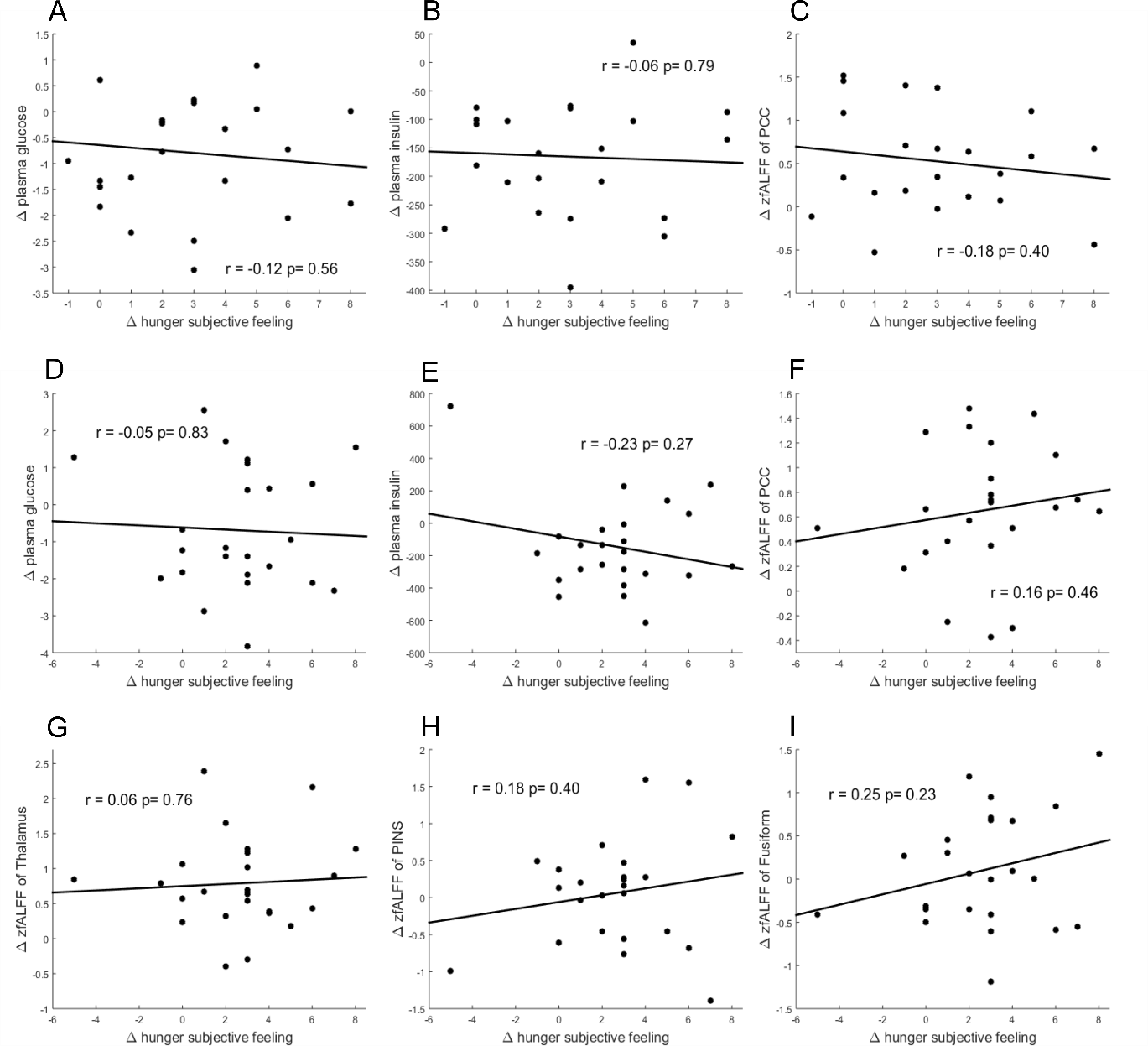 |
| --- |
| Figure S3. Scatter plots illustrate the correlations between changes subjective feeling of hunger with changes plasma glucose and insulin as well as with changes in brain activity (i.e. clusters that survived the cluster-significance test). (A)-(C) represent the associations before glucose administration. (D)-(I) represent the associations after glucose administration. ∆ refers to the difference between hunger and satiety. |
